# A critical moment in machine learning in medicine: on reproducible and interpretable learning

**DOI:** 10.1007/s00701-024-05892-8

**Published:** 2024-01-16

**Authors:** Olga Ciobanu-Caraus, Anatol Aicher, Julius M. Kernbach, Luca Regli, Carlo Serra, Victor E. Staartjes

**Affiliations:** 1 Machine Intelligence in Clinical Neuroscience & Microsurgical Neuroanatomy (MICN) Laboratory, Department of Neurosurgery, Clinical Neuroscience Center, University Hospital Zurich, University of Zurich, Zurich, Switzerland; 2 Department of Neuroradiology, University Hospital Heidelberg, Heidelberg, Germany

**Keywords:** Machine learning, Reproducibility, Interpretability, Methodology

## Abstract

Over the past two decades, advances in computational power and data availability combined with increased accessibility to pre-trained models have led to an exponential rise in machine learning (ML) publications. While ML may have the potential to transform healthcare, this sharp increase in ML research output without focus on methodological rigor and standard reporting guidelines has fueled a reproducibility crisis. In addition, the rapidly growing complexity of these models compromises their interpretability, which currently impedes their successful and widespread clinical adoption. In medicine, where failure of such models may have severe implications for patients’ health, the high requirements for accuracy, robustness, and interpretability confront ML researchers with a unique set of challenges. In this review, we discuss the semantics of reproducibility and interpretability, as well as related issues and challenges, and outline possible solutions to counteracting the “black box”. To foster reproducibility, standard reporting guidelines need to be further developed and data or code sharing encouraged. Editors and reviewers may equally play a critical role by establishing high methodological standards and thus preventing the dissemination of low-quality ML publications. To foster interpretable learning, the use of simpler models more suitable for medical data can inform the clinician how results are generated based on input data. Model-agnostic explanation tools, sensitivity analysis, and hidden layer representations constitute further promising approaches to increase interpretability. Balancing model performance and interpretability are important to ensure clinical applicability. We have now reached a critical moment for ML in medicine, where addressing these issues and implementing appropriate solutions will be vital for the future evolution of the field.

## Introduction

The evolution of machine learning (ML) has opened new frontiers in the analysis of highly-dimensional data that expands in some cases far beyond the possibilities of traditional statistical modeling [44, 57, 62]. Over the past two decades, ML applications have seen an exponential rise at an accelerating pace of innovation, fueled by the advances in computational power, data availability, and dimensionality [2]. The current surge in popularity of ML as well as the low threshold towards getting started with ML-based research through the availability of open-access pre-trained models and coding libraries have further democratized ML. With an exponential number of publications on ML in medicine, this raises serious potential concerns about how methodological rigor and reproducibility can be upheld—especially since most clinical journals, editors, reviewers, and readers are not necessarily prepared to judge whether a ML manuscript actually presents a valid application [60]. Especially the reproducibility—and science depends on reproducing experiments to verify results—is often not addressed, as a majority of ML publications do not report the necessary information to understand and validate exactly what was done. Another issue is the rapidly growing complexity of applied techniques, which compromises their interpretability (“black box” issue). This frequently impedes their successful widespread clinical adoption and has given rise to the quest for reproducible and interpretable ML [30]. While several ML models have been successfully developed and utilized in various areas outside of medicine, the implementation of such models in clinical medicine is still limited [30]. This reluctance to translate ML models into healthcare also stems from the fact that their results may have direct implications for patient well-being [59]. Consequently, the high requirements for performance, robustness, and interpretability pose specific challenges for ML developers and health professionals [26]. The vastly heterogeneous properties of the published models of the current ML landscape have fueled a reproducibility and interpretability crisis which constitutes a considerable risk for the liability and credibility of ML in medicine [20, 21].

We have now reached a critical moment for ML in medicine, where dedicating efforts to resolving these issues will be vital for the future evolution of the field. We must uphold methodological standards, as we do for other fields of medical research such as clinical trials. This review therefore aims to critically reflect on current issues and challenges related to reproducibility and interpretability of ML models, highlight possible solutions and give an outlook on future directions of the field.

## Reproducibility

### Definition

Reproducibility and replicability are scientific principles and should be fought for especially in medical ML. In addition, they represent a prerequisite for a model to be interpretable—as only interpretations of rigorous models are clinically useful [1]. Reproducibility refers to the ability of an independent research group to reproduce the results of an original study using the same data and code [23, 47]. Intuitively, the term reproducibility is often synonymous with technical reproducibility; however, reproducibility in a broader sense also encompasses statistical and conceptual reproducibility [45]. Statistical reproducibility denotes that a research group is able to reach similar results in a resampled dataset, also called internal validity [9]. Conceptual reproducibility describes that an independent group is able to verify the results using the same code but based on different data, frequently referred to as replicability and therefore closely related to the notion of external validity [1, 9, 45, 53].

### Issues and challenges

In comparison to general ML domains where researchers adopted fairly radical notions of open science and transparency, ML model developers in healthcare face a unique set of challenges which are a result of the inherent nature of healthcare data, regulations, and systems [6]. Technical reproducibility depends on data and code release; however, sharing health data is often highly problematic due to strict data protection regulations. By their inherent nature, health datasets tend to be relatively small in terms of number of observations, noisy, of high dimensionality, and often suffer from irregular sampling, therefore limiting statistical reproducibility [45]. In addition, patient populations display individual differences in treatment response, diversifying outcomes in a way that complicates outcome predictions [5]. Furthermore, datasets are frequently derived from single centers, limiting the generalizability of ML models as the cohort represented by the dataset is often narrower than the population it is intended to reflect [45]. A literature review of 511 articles presented at ML conferences from 2017 to 2019 concluded that of all ML papers in healthcare, only 55% used publicly available datasets, only 21% shared their analysis code, only 44% of papers reported variance of their performance metrics, and only 23% of papers used multi-institutional datasets [45].

Much more than the amount of input data, it is its quality that ultimately determines the performance of a ML model. Missing, inconsistent, inaccurate, or biased data may significantly limit the predictive accuracy [14, 48]. Although ML methods represent valuable and powerful tools for data analysis, they may also suffer from statistical vulnerability [30]. ML models learn patterns of data to generate decisions—and will therefore also inherit concealed bias and inaccuracies of the input data [14]. Various forms of data leakage—a phenomenon where information from a training set contains data from the testing set—may introduce additional bias, leading to overfitting of the ML models and compromising their reproducibility [33]. A systematic review of bias assessments analyzing over 2000 clinical prediction models found that a substantial proportion of these—ranging from one quarter to two thirds—displayed a high risk of bias based on either their statistical analysis, outcome definition, or participant selection [3, 66]. With regard to reporting predictive accuracy, the choice of an appropriate measure to report predictive accuracy represents an additional challenge as one metric may not translate into another, and not every metric be interpretable in a clinically meaningful way [12].

Whereas randomized controlled trials (RCTs) and observational studies are generally subject to methodological rigor and undergo intense scrutiny to ensure high standards of the stability of analyses and adequate reporting of results, such efforts have not been equally mirrored in the research of ML models [6]. As an aggravating factor, the responsibility to identify potentially irreproducible or low-quality ML models remains in the hands of peer reviewers of medical journals, who may not always be well-equipped to scrutinize these models [21]. Even after the fact, readers of medical journals often cannot critically appraise published ML articles in the same way of standardized RCTs.

### A framework for optimizing reproducibility

Various solutions have been proposed to address these issues. Shared and auditable large-scale multi-institutional, multi-national data repositories as well as shared code and guidance on best practices have been shown to foster reproducibility and provide more generalizable results [10, 50]. Publicly available datasets such as MIMIC-III [32], Phillips eICU [55], and the UK Biobank [61] represent promising examples, yet comparable efforts to create more of such datasets are required. Datasets with meticulous descriptions of their contents, details on incompleteness, inconsistency, confounders and biases, and missing data are crucial to enable standardized data collection and clarify their usage for subsequent analyses [52].

Concerning the safe release of data, numerous technological solutions are being developed to mitigate privacy issues [36]. Generating synthetic data that resembles original health data may allow researchers to share their code with full end-to-end realization of their pipeline [65]. In homomorphic encryption approaches, computations are performed on data that has been previously encrypted using cryptographic techniques [8]. Federated learning hides privacy-related data by sharing only globally averaged updated parameters on a server which are provided by learned parameters of local models at each client’s site—avoiding data sharing altogether [11].

As is already common practice for RCTs and numerous observational studies, pre-registering studies, specifying a priori hypotheses and designing a precise statistical plan would help uphold the methodological accuracy of ML studies [38]. Standard reporting guidelines including TRIPOD, CONSORT, and SPRINT are increasingly adapted for ML and AI applications [12, 13]. In addition to adherence to these guidelines, efforts should be directed towards the development and dissemination of best practices for ML analyses [27]. Given the limited number of experienced ML researchers in the medical community, it also lies in the responsibility of journal to ensure reviewers with the appropriate academic background are recruited—for example, dedicated ML editors and reviewers should be assigned by each journal. Quality assessment checklists and guidelines such as ROBUST-ML [2] or MI-CLAIM [49] may serve as valuable tools for reviewers to enable a systematic evaluation of the quality of ML studies. MI-CLAIM (minimum information about clinical artificial intelligence modeling) [49] is a six-part-checklist to ensure transparency and interpretability of ML studies by establishing standard minimum requirements for study design, regarding the clinical setting, performance measures, population composition, and standard reference for comparison of a ML model. Furthermore, MI-CLAIM recommends partitioning of the dataset into a training and testing cohort, gives recommendations on optimization and model selection, performance evaluation, and sets standards for reproducibility [49]. Poldrack et al. [54] proposed a framework of best practices to ensure accurate reporting of estimates of predictive validity, help quantify predictive accuracy, and prevent misinterpreting evidence for correlation with actual prediction: analyses should be based on a training cohort of at least several hundred observations. Moreover, all operations applied to the data should be included in cross-validation procedures, and *k*-fold cross-validation with a low *k* should be used preferably (as opposed to the other extreme being leave-one-out-cross-validation). In-sample model fit indices should not serve as measure for predictive accuracy, and rather multiple measures should be reported. In comparison with a correlation coefficient, a coefficient of determination should be preferably used. Applying these best practices may effectively combat various issues that are encountered in prediction modeling, increase predictive performance, and guarantee a higher generalizability of results [54].

## Interpretability

### Definition

High reproducibility and a robust performance are prerequisites for clinical implementation. However, to truly support clinical decision-making and gain credibility, a ML model needs to also become interpretable—in other words, clinicians need to understand how their ML models come to their decisions [16]. Interpretability refers to the ability to trace back how a ML model generates its results and is frequently interchangeably used with the term explainability [22, 59].

### Issues and challenges

ML algorithms can be classified as interpretable or non-interpretable (“black box”) models, by their respective architecture. While interpretable ML models generally appear to be more transparent in their underlying explanatory structures, non-interpretable ML models may frequently reach higher performance metrics. Balancing performance with interpretability is therefore of paramount importance to ensure the translation and clinical adoption of ML models [43].

Especially in the medical field, this balance is primarily ethical in nature [56]: providing solid explanations for the behavior of an algorithm for diagnosis, treatment recommendations, disease prognosis, or mortality prediction while ensuring a high degree of accuracy is a fundamental prerequisite for the social acceptance and trustworthiness of a model—not only because incorrect results may potentially have real-world consequences for the well-being of patients [28] but also because it is not clearly regulated who takes legal responsibility in the case of adverse events [4]. Understanding the relationship between input and output of the model is therefore essential both for the clinician to be able to make informed treatment decisions and for the patient to be able to give informed consent [64]. As the structural architecture of ML evolves to highly complex non-linear architectures such as convolutional neural networks, the behavior of the algorithm and the underlying causal relationships leading to a specific result become increasingly difficult to explain [40]. While most medical ML should be tackled using natively interpretable, simple models, in medical imaging applications, deep learning models can be highly useful, although they lack in interpretability [34]. While feature selection and engineering are key properties of other ML techniques, deep learning can automatically learn useful representation of data and sometimes reach superior performance [29, 68]. Automatically, extracted features may easily mount up to thousands of variables which are extraordinarily difficult for clinicians to interpret [29].

### De-black-boxing ML

Black box models that do not offer native methods for interpretation require special attention. For ML to become interpretable, two major structural aspects of the model need to be explained in a transparent, humanly understandable way: first, the logic of the model (model-based explanations), and second, the causal relationships between input and output of a model (results explanations) [24].

Model-agnostic explanation methods such as SHapley Additive exPlanation (SHAP) or Local Interpretable Model-agnostic Explanation (LIME) represent two prominent interactive techniques for model behavior [35]. SHAP is a commonly used approach which quantifies the individual contribution of a feature value (Shapley value) to the difference between the actual and the average prediction of model, detailed as relative distribution among features [41]. LIME can be used to explain how individual features lead to prediction probabilities by approximating it locally with an interpretable model [58]. For this, LIME perturbs sampled training data for classifiers and assesses how changes in the features affect the results of a model [58]. However, this technique is an extremely time-consuming approach which in addition is also exceptionally prone to bias [18].

As for results explanation, the choice of an appropriate explanation method largely depends on the internal architecture of the ML mode. Uniform manifold approximation and projection for dimension reduction (UMAP) has become the established method for feature space visualization while gradient-weighted class activation mapping (Grad-CAM) is frequently used to explain deep neural networks [7, 63]. Unraveling DL models by providing explanatory graphs for the knowledge hierarchy concealed in the convolution-layers of a convolutional neural network, so-called hidden layer representation, constitute a promising approach to improving interpretability [67, 69].

However, many applications of ML in medicine do not provide enough input data for sensible use of such more complex architectures, and in most cases architectures such as generalized linear models, decision trees, or random forests are more appropriate—these techniques natively support interpretation (as they provide coefficients, visualized trees, or Gini importance, for example) [42].

In general, simple models such as nomograms or decision trees favor clinical applicability as they are widely understood and more easily applicable. In other words, the first step in ensuring interpretability should always be asking “do I really need a complex model here?”. Another option are graphical calculation devices named “nomograms”, used to explain logistic regression-based analyses, as is already common in oncology applications [31]. Decision trees model nonlinear effects and frequently detail feature importance scores, making them highly interpretable—provided a shallow tree depth [15].

Sensitivity analysis may help to assess how changes in input feature impact the predictive performance of a DL model [39]. In this context, heat maps are a valuable tool to visualize the importance of each pixel for a prediction task and may optimize a convolutional neural network training approach [43]. For example, heat maps detailing the Z-score difference of each radiomic feature between the training and the validation data set may be used to evaluate consistency of radiomics features [37].

Especially in medicine, where failure of a model may adversely affect patient health, constant automatic and human-in-the-loop evaluation of its interpretability is required to test and optimize the performance of a model in a clinical setting. Using applications in their daily practice, clinicians can determine the performance model by comparing the explanation of a model with their own explanation for a decision [17]. The most trivial form of human-based evaluations constitute studies which compare the accuracy of decisions made by clinicians with or without interpretable ML; however, they are highly susceptible to interobserver variability caused by subjectivity and personal preferences [19]. To combat these potential forms of bias, multiple readers should be employed on a high number of diverse cases [19]. Forward and counterfactual simulation studies may aid in the objective assessment of interpretability to capture whether the clinician comprehends the underlying rationale behind the prediction [18]. In forward simulation, a reader is supposed to predict model output based on given input data. In counterfactual simulation, a clinician should predict the model result given a change to the input data [25]. Furthermore, human-subject involvement in the evaluation of explanation methods has been proposed in a feedback or feed-forward setting [46]. In a feedback setting, clinicians provide feedback on explanations which then used to quantify the quality of explanations [46]. In a feed-forward setting, clinicians suggest examples for explanations which serve as a reference for the explanations of the ML model [46].

## Conclusion

Accessibility to ML techniques and the explosion in medical publishing overall have fueled the current “hype” of medical ML, which has certainly led to some interesting advancements, but also begs the question for how we should gatekeep proper techniques, rigorous methodology [45]. Many of the currently published ML models in the medical literature do not correspond to the “state-of-the-art—not only in terms of general methodology, but increasingly also in terms of lack of reproducibility and interpretability. As we outline in this review, these two points are crucial for the success of introducing ML into clinical practice. Raising awareness to these issues, providing solutions and establishing rigorous standards for ML research will be of utmost importance to de-stigmatize black-box-like models and restore the credibility and legitimacy of ML in medicine [51]. Data and code sharing (if necessary using approaches like federated learning), proper reporting according to guidelines, installing dedicated ML expert reviewers, applying simple and natively interpretable models where possible, or using post hoc techniques to enable interpretation of complex models where this is not: these gatekeeping steps will be critical to ensure that ML—like any scientific method – is applied correctly and does not produce misleading or even dangerous results [18].
